# Clinical Experience of Proteasome Inhibitor Bortezomib Regarding Efficacy and Safety in Severe Systemic Lupus Erythematosus: A Nationwide Study

**DOI:** 10.3389/fimmu.2021.756941

**Published:** 2021-10-01

**Authors:** Tomas Walhelm, Iva Gunnarsson, Rebecca Heijke, Dag Leonard, Estelle Trysberg, Per Eriksson, Christopher Sjöwall

**Affiliations:** ^1^ Department of Biomedical and Clinical Sciences, Division of Inflammation and Infection/Rheumatology, Linköping University, Linköping, Sweden; ^2^ Department of Medicine Solna, Division of Rheumatology, Karolinska Institute, and Rheumatology, Karolinska University Hospital, Stockholm, Sweden; ^3^ Department of Internal Medicine, Jönköping, Sweden; ^4^ Department of Medical Sciences and Science for Life Laboratory, Uppsala University, Uppsala, Sweden; ^5^ Department of Rheumatology and Inflammation Research, University of Gothenburg, Göteborg, Sweden

**Keywords:** bortezomib (BTZ), systemic lupus - erythematosus, Lupus nephritis (LN), adverse (side) effects, antinuclear antibodies, clinical efficacy analysis, observational study

## Abstract

As treatment options in advanced systematic lupus erythematosus (SLE) are limited, there is an urgent need for new and effective therapeutic alternatives for selected cases with severe disease. Bortezomib (BTZ) is a specific, reversible, inhibitor of the 20S subunit of the proteasome. Herein, we report clinical experience regarding efficacy and safety from all patients receiving BTZ as therapy for SLE in Sweden during the years 2014−2020. 8 females and 4 males were included with a mean disease duration at BTZ initiation of 8.8 years (range 0.7–20 years). Renal involvement was the main target for BTZ. Reduction of global disease activity was recorded by decreasing SLEDAI-2K scores over time and remained significantly reduced at the 6-month (p=0.007) and the 12-month (p=0.008) follow-up visits. From BTZ initiation, complement protein 3 (C3) levels increased significantly after the 2^nd^ treatment cycle (p=0.05), the 6-month (p=0.03) and the 12-month (p=0.04) follow-up visits. The urine albumin/creatinine ratio declined over time and reached significance at the 6-month (p=0.008) and the 12-month follow-up visits (p=0.004). Seroconversion of anti-dsDNA (27%), anti-C1q (50%) and anti-Sm (67%) was observed. 6 of 12 patients experienced at least one side-effect during follow-up, whereof the most common adverse events were infections. Safety parameters (C-reactive protein, blood cell counts) mainly remained stable over time. To conclude, we report favorable therapeutic effects of BTZ used in combination with corticosteroids in a majority of patients with severe SLE manifestations irresponsive to conventional immunosuppressive agents. Reduction of proteinuria was observed over time as well as seroconversion of some autoantibody specificities. In most patients, tolerance was acceptable but mild adverse events was not uncommon. Special attention should be paid to infections and hypogammaglobinemia.

## Introduction

Despite advances in treatment strategies leading to an improved prognosis, several challenges and unmet needs remain for patients living with systemic lupus erythematosus (SLE) (1, 2). In sharp contrast to other rheumatic diseases, treatment options in advanced SLE are limited. Subsequently, there is an urgent need for new and effective therapeutic alternatives for selected cases with severe disease. After many years of disappointing results from randomized clinical trials, recent outcomes of phase III trials on, *e.g.* anifrolumab and voclosporin, raise hope for clinicians and patients with SLE (3). However, many patients still experience refractory disease.

B cells have a prominent role in the pathogenesis of SLE as they mediate inflammation *via* production of a broad spectrum of autoantibodies directed against nuclear or cytoplasmic constituents and plasma proteins (4). Arguments for a pathogenic role include the fact that autoantibodies, such as anti-Smith (Sm) and anti-double stranded DNA (dsDNA), are associated with the clinical presentation of the disease, and the level of anti-dsDNA frequently correlates with SLE disease activity (5, 6). Today, different strategies are used to target the various stages of B cell development and, besides clinical disease activity, autoantibody levels are frequently used as surrogate markers of efficacy of the B cell-directed therapies (7). Most of these immunosuppressants, commonly used in combination with corticosteroids, primarily exert their therapeutic effects on B cells, plasmablasts and/or short-lived plasma cells (8, 9). However, to achieve effects also on the long-lived plasma cells, the available alternatives are autologous stem cell transplantation, atacicept [which blocks both the B cell activating factor (BAFF) and a proliferation-inducing ligand [APRIL)] and proteasome inhibition (9–11).

Bortezomib (BTZ) is a specific, reversible, and cell permeable dipeptide boronic acid inhibitor of the chymotryptic activity of the 20S subunit of the proteasome (12). Plasma cells are vulnerable to proteasome inhibition as it causes accumulation of defective immunoglobulin chains, resulting in endoplasmic reticulum stress, misfolded protein response, and subsequent apoptosis of plasma cells (13, 14). Long-lived plasma cells are significant antibody producers, they are highly sensitive to proteasome inhibition. In addition, proteasome inhibitors also effectively function as inhibitors of the production of pro-inflammatory cytokines through the regulation of NF-κB activation (7, 11).

Besides multiple myeloma and mantle cell lymphoma where BTZ is approved since the beginning of this century, advantageous effect of BTZ was demonstrated in German patients with renal and extra-renal severe SLE some years ago (15). Later, positive experience of BTZ in refractory lupus nephritis (LN) was reported from Spain and China although side-effects such as infections and neuropathy led to discontinuation in some cases (16, 17). Furthermore, the autoantibody repertoires of patients with SLE receiving BTZ or rituximab (RTX) ± belimumab (BLM) have been shown to differ illustrating the drugs’ separate mechanism of action and highlight their impact on different B cell subsets (18). In a Japanese multicenter double-blind randomized controlled phase II trial including 14 patients, favorable clinical effects were observed on an individual level in some patients but also adverse events like fever, liver dysfunction and hypersensitivity reactions (19). Nevertheless, the study overall could not demonstrate efficacy of BTZ.

In 2014, the first Swedish patient with SLE was treated with BTZ. This female had life-threating disease characterized by proliferative LN, which was resistant to both cyclophosphamide (CYC) and RTX, and concomitant diffuse alveolar bleeding (20). Since then, another 11 patients with severe lupus manifestations have been started on BTZ at rheumatology practices in Sweden. Herein, we describe clinical efficacy and safety data from all Swedish patients receiving BTZ as therapy for SLE during the years 2014−2020. To our knowledge, high-quality nationwide real-life data of BTZ in SLE has previously not been communicated.

## Materials and Methods

### Patients

Patient data were retrieved from all Rheumatology practices at Swedish University hospitals; 4 out of 7 tertiary referral centers offering high-specialized rheumatology health-care services (Göteborg, Linköping, Stockholm and Uppsala) and one county hospital (Jönköping) had experience of using BTZ for SLE during the years 2014−2020. All subjects eligible for BTZ treatment had been classified with SLE according to the 1997 American College of Rheumatology (ACR) criteria (21). Clinical characteristics of the included patients are detailed in **Table 1**.

**Table 1:** Characteristics of the included patients (n = 12) at the initiation of BTZ treatment.

Patient characteristics	
***Background variables***	**Mean (range) or %**
Females	66.7
Age at SLE onset (years)	30.1 (6−71)
SLE duration (years)	8.8 (0.7−20)
SLEDAI-2K (score)	14.4 (10−20)
SDI (score)	0.8 (0−3)
Body Mass Index (kg/m^2^)	30.9 (20.2−43.0)
Caucasian ethnicity	58.3
Number of fulfilled ACR-97 criteria	6.7 (4−9)
Antiphospholipid syndrome	1 (8.3)
***Clinical phenotypes (ACR-97 definitions)***	**n (%)**
Malar rash	7 (58.3)
Discoid lupus	2 (16.7)
Photosensitivity	5 (41.7)
Oral ulcers	4 (33.3)
Arthritis	10 (83.3)
Serositis	4 (33.3)
Renal disorder	11 (91.7)
Neurological disorder	1 (8.3)
Hematological disorder	12 (100)
Immunological disorder	12 (100)
Anti-nuclear antibody	12 (100)
***Target organ system***	**n (%)**
Renal	11 (91.7)
Histopathology^1^	**n (%)**
Class III	3 (27.3)
Class IV	5 (45.5)
Class V	2 (18.2)
No biopsy available	1 (9.1)
Central nervous system (transverse myelitis)	1 (8.3)
Liver (autoimmune hepatitis)^2,3^	1 (8.3)
Lung (diffuse alveolar bleeding)^2^	1 (8.3)

ACR, American College of Rheumatology; SLEDAI-2K, Systemic Lupus Erythematosus disease activity index 2000; SDI, SLICC/ACR damage index.

^1^Histopathology staged according to the International Society of Nephrology/Renal Pathology Society (ISN/RPS) classification for LN (22).

^2^Concomitant with active Class IV lupus nephritis.

^3^Liver biopsy showed inflammation grade 3−4 and fibrosis stage 3 according to Batts & Ludwig (23).

### Treatment Regimen

**Figure 1:**
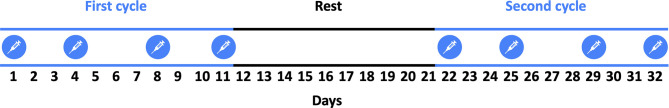
As shown in the schematic illustration, the provided dosage of bortezomib (BTZ) was 1.3 mg/m^2^ subcutaneously on day 1, 4, 8 and 11 along with dexamethasone (20−50 mg), followed by 10 days of rest before start of the next treatment cycle.

The provided dosage of BTZ was 1.3 mg/m^2^ subcutaneously on day 1, 4, 8 and 11 along with dexamethasone (20−50 mg), followed by 10 days of rest before start of the next treatment cycle as illustrated in **Figure 1** (15). Two or three BTZ cycles were administrated for all but one patient. Data on disease-modifying anti-rheumatic drugs (DMARDs) and prednisolone dose used before BTZ initiation as well as concomitant immunomodulatory treatment during and following BTZ were collected.

### Clinical Evaluation

SLE disease activity was assessed using the Physician’s Global Assessment (PGA, graded 0−100) and the Systemic Lupus Erythematosus disease activity index 2000 (SLEDAI-2K) (24, 25). Acquired organ damage, required to have been persistent for at least 6 months, was recorded by the Systemic Lupus International Collaborating Clinics (SLICC)/ACR damage index (SDI) encompassing damage in 12 defined organ systems (26).

### Laboratory Measurements

Safety was continuously monitored by blood cell counts (erythrocytes, leukocytes, lymphocytes, neutrophils, and platelets) and C-reactive protein (CRP). Inflammatory and serological disease activity were followed by the autoantibodies (anti-dsDNA, anti-Sm, anti-C1q), erythrocyte sedimentation rate (ESR), and plasma analyses of albumin, immunoglobulin G (IgG), complement protein 3 (C3) and 4 (C4) according to clinical routine at the treating hospitals. Renal function was monitored by plasma creatinine, estimated glomerular filtration rate (eGFR) based on plasma creatinine, according to the MDRD 4-Variable Equation (27), and the urine albumin/creatinine ratio.

### Histopathology

Renal (n=10) and liver (n=1) biopsies were performed by percutaneous ultrasonography-guided puncture in accordance with a standard protocol. The renal tissue obtained was staged according to the International Society of Nephrology/Renal Pathology Society (ISN/RPS) classification for LN (22). The liver biopsy was assessed according to the standardized semi-quantitative histologic scoring system for hepatitis developed by Batts and Ludwig (23).

### Statistics

Wilcoxon’s matched-pairs test was used for comparing paired patient data. Associations between adverse events (binary variable) and hypogammaglobulinemia (binary variable) were examined with Fisher’s exact test. Statistical analyses were performed using the SPSS software version 27.0.0.0 (SPSS Inc., Chicago, IL, USA) and Prism 9 (GraphPad Software Inc., La Jolla, USA) for construction of graphs. All laboratory data graphs are showing means with standard deviation. P-values ≤0.05 were considered statistically significant.

## Results

### Subjects Treated With BTZ

In total, 8 females and 4 males with a disease duration ranging from 8 months to 20 years (mean 8.8 years) were considered eligible for BTZ and included in the follow-up. The mean age at BTZ initiation was 38.5 years and the global disease activity assessed by SLEDAI-2K was 14.4 (mean score). The target organ system for BTZ treatment was kidney (n=11) with proliferative (Class III and IV) histopathology dominating (n=8). One of the patients with proliferative LN had concomitant active autoimmune hepatitis requiring immunosuppression. In addition, one female had active central nervous system (CNS) involvement manifested by transverse myelitis without LN. 10 of 12 patients had an inadequate response to CYC and/or RTX ahead of BTZ initiation (**Table 2**).

**Table 2:** Individual descriptions of the 12 included patients.

Patient number and gender	Age at BTZ initiation	BTZ cycles	Prednisolone, daily dose (mg)*		DMARDs ahead of BTZ initiation**	Concomitant immunomodulatory treatment	Immunomodulatory treatment following BTZ***	Adverse events
			Before	After				
1/F	26	2	5	5	HCQ, MMF, RTX	HCQ	HCQ, MMF	None reported
2/M	38	0.75	7.5	20	CYC, HCQ	HCQ	ABA, AZA, HCQ	Massive edema, viral infection
3/M	37	2	7.5	5	MMF	HCQ	BLM, HCQ, MMF	None reported
4/F	41 43	2 2	10 5	10 5	BLM, CYC, HCQ, MMF, RTX	HCQ	BLM, HCQ, MMF	Otitis media, lower UTI
5/M	71	2	20	10	MMF, RTX		MMF	None reported
6/F	40	2	10	7.5	CYC, HCQ, MMF, RTX	HCQ	BLM, MMF, HCQ	None reported
7/F	29	2	10	10	CsA, HCQ, RTX	HCQ	BLM, HCQ	None reported
8/F	36	3	25	10	HCQ, MMF	HCQ	HCQ, MMF	None reported
9/M	21	2	15	15	HCQ, MMF, RTX	HCQ	HCQ, MMF, TAC	Renal anemia, diarrhea, hyperkalemia, hypernatremia, elevated liver enzymes
10/F	37	3	15	10	HCQ, RTX	HCQ	ABA, BLM, HCQ, MMF, PE	Pulmonary embolism
11/F	59	2	40	20	CYC, MMF, MTX, RTX	PE	BLM, HCQ	Cryptogenic organizing pneumonia
12/F	29	3	20	10	BLM, CsA, CYC, HCQ, MMF, RTX		MMF	Fever

*30-day average value, **12 months before the 1^st^ BTZ cycle, ***12 months after the last BTZ cycle.

ABA, abatacept; BLM, belimumab; BTZ, bortezomib; CsA, cyclosporine A; CYC, cyclophosphamide; DMARDs, disease-modifying anti-rheumatic drugs; F, female; HCQ, hydroxychloroquine; M, male; MMF, mycophenolate mofetil; MTX, methotrexate; RTX, Rituximab; TAC, tacrolimus; PE, plasma exchange; UTI, urinary tract infection.

### BTZ Cycles and Maintenance Therapy

Of the included 12 subjects, 9 patients received two cycles of BTZ and two individuals three cycles. In the last patient, BTZ was discontinued before the 1^st^ cycle was completed due to adverse effects (see below). One patient (Nr 4, see **Table 2**) received BTZ therapy at two occasions, with two cycles given both times.

Data of concomitant immunomodulatory treatment ahead of BTZ and following immunomodulatory treatment post-BTZ are shown in **Table 2**. Hydroxychloroquine (HCQ) was administrated during BTZ treatment in 9 of the 12 subjects. Four patients used combined mycophenolate mofetil (MMF) and BLM after completed BTZ cycles, whereas 10 of 12 subjects were prescribed HCQ in combination with other DMARDs. The mean daily prednisolone dosage at initiation of BTZ was 14.6 mg (5−40 mg); the prednisolone dose 30 days after ended BTZ treatment was 10.6 mg (5−20 mg).

### Efficacy

**Figure 2:**
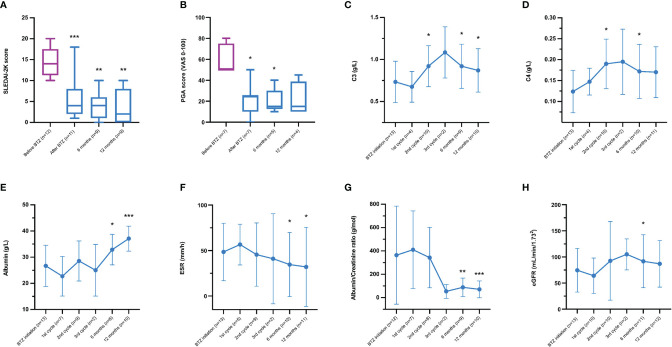
**(A–H)** Clinical evolution and laboratory efficacy data over time from bortezomib (BTZ) initiation to the 12-month follow-up visit; **(A)** Systemic Lupus Erythematosus disease activity index 2000 (SLEDAI-2K), **(B)** Physician’s Global Assessment graded (PGA) by visual analogue scale (VAS) 0−100 mm, **(C)** complement protein 3 (C3), **(D)** complement protein 4 (C4), **(E)** albumin in plasma, **(F)** erythrocyte sedimentation rate (ESR), **(G)** urine albumin/creatinine ratio, and **(H)** estimated glomerular filtration rate (eGFR) based on plasma creatinine, according to the MDRD 4-Variable Equation (27). Since one patient (without genetic deficiency of C1q, C3 or C4) received BTZ therapy at two occasions, the number of observations was 13 at some time-points. The graphs represent the mean and standard deviation; significances correspond to comparisons with baseline values. *p < 0.05. **p < 0.01 and ***p < 0.005.

Disease activity assessments are shown in **Figures 2A, B**. As compared to BTZ initiation, SLEDAI-2K scores (mean 14.4) were significantly reduced (i) at the end of BTZ treatment (mean 6.1, p=0.003), (ii) the 6-month follow-up visit (mean 4.0, p=0.007) and (iii) the 12-month follow-up visit (mean 4.0, p=0.008). By assessing disease activity with PGA, a significant reduction was observed at the end of the last treatment cycle (p=0.03) as well as the 6-month follow-up visit (p=0.04) compared to start of BTZ.

Complement proteins during follow-up are illustrated in **Figures 2C, D**. From BTZ initiation, C3 levels increased significantly after the 2^nd^ cycle (p=0.05), the 6-month follow-up visit (p=0.03) and the 12-month follow-up visit (p=0.04). C4 levels increased significantly but only after the 2^nd^ cycle (p=0.03) and at the 6-month follow-up visit (p=0.03) compared to BTZ start.

Plasma albumin (**Figure 2E**) increased over time at the 6-month follow-up visit (p=0.02) and at the 12-month follow-up visit (p=0.005). ESR (**Figure 2F**) decreased over time at the 6-month follow-up visit (p=0.03) and at the 12-month follow-up visit (p=0.05). The urine albumin/creatinine ratio (**Figure 2G**) declined over time, reaching statistical significance at the 6-month follow-up visit (p=0.008) as well as at the 12-month follow-up visit (p=0.004) post-BTZ treatment. eGFR (**Figure 2H**) was significantly improved at the 6-month follow-up visit (p=0.05).

Anti-dsDNA antibodies were positive in 11 of 12 subjects (91.7%) at start; 3/11 (27.3%) had seroconverted at the last follow-up. Anti-C1q antibodies were positive in 4 of 10 subjects (40%) at start; 2/4 (50%) had seroconverted at the last follow-up. Anti-Sm antibodies were positive in 3 of 11 subjects (27.3%) at start; 2/3 (66.7%) had seroconverted at the last follow-up.

### Safety

As demonstrated in **Table 2**, 6 of 12 patients experienced at least one side-effect during follow-up. The most common adverse events were infections. One of four infections were severe and led to hospitalization. One individual did not complete the 1^st^ cycle due to fever (viral infection) and emerging nephrotic syndrome with subsequent edema. The other patients fulfilled at least two cycles. Neuropathy was not reported in any subject.

Plasma IgG levels decreased during the BTZ cycles (median values: 9.1 g/L pre-BTZ *vs*. 7.8 g/L post-BTZ; p=0.008). 5/12 (42%) patients developed hypogammaglobulinemia (<6.7 g/L) during the BTZ treatment. 3 of 5 individuals who developed hypogammaglobulinemia experienced adverse events compared to 3 of 7 of those with IgG levels within the reference interval (not significant).

**Figure 3:**
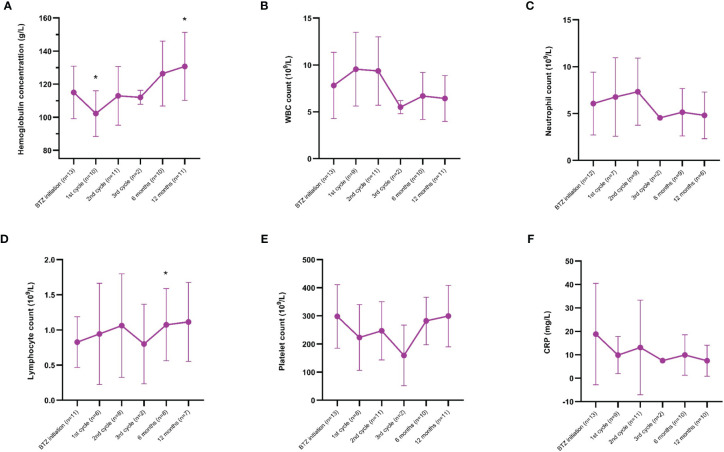
**(A−F)** Laboratory safety data over time from bortezomib (BTZ) initiation to the 12-month follow-up visit; **(A)** hemoglobin, **(B)** white blood cell (WBC) count, **(C)** neutrophil count, **(D)** lymphocyte count, **(E)** platelet count, and **(F)** C-reactive protein (CRP). Since one patient received BTZ therapy at two occasions, the number of observations was 13 at some time-points. The graphs represent the mean and standard deviation; significances correspond to comparisons with baseline values. *p < 0.05.

CRP and blood cell counts, including hemoglobin, leukocytes, neutrophils, lymphocytes and platelets, are illustrated in **Figure 3**. The hemoglobin concentration decreased after the 1^st^ cycle (p=0.04), followed by a significant increase at the 12-month follow-up visit (p=0.03). The lymphocyte count increased over time at the 6-month follow-up visit (p=0.04). Leukocyte, neutrophil and platelet counts, as well as CRP levels, did not change significantly over time.

## Discussion

The data presented originate from clinical follow-up of patients at Swedish academic practices and monitored by a limited number of SLE specialists. The efficacy of BTZ was continuously evaluated using a validated global disease activity scoring system. Overall, reduced SLEDAI-2K scores were observed early and remained reduced at the 6- and 12-month follow-up. Our findings are in line with experiences from both European and Chinese centers (15–17). Furthermore, we found that complement consumption, mirroring serological disease activity at BTZ initiation, tended to normalize over time.

Due to different immunoassays used for anti-dsDNA analysis at the four University units, we were not able to report reliable data on longitudinal autoantibody levels. However, seroconversion of anti-dsDNA (27.3%), anti-C1q (50%) and anti-Sm (66.7%) were observed. Reduction of several autoantibody specificities and antiphospholipid antibodies during BLM therapy have been reported (28–30), but it should be emphasized that anti-Sm is mainly produced by long-lived plasma cells which usually are not reached by drugs like CYC, RTX or BLM (31). The finding of seroconversion of anti-Sm in up to two-thirds of the SLE patients after BTZ could thus reflect a deeper depletion than with CYC/RTX. These observations partially contrast the findings reported by van Dam et al. who described significant decrease of anti-dsDNA during BTZ therapy whereas anti-C1q antibodies essentially remained unaffected (18). However, in the Japanese multicenter double-blind randomized controlled phase II trial of BTZ in SLE, reduction of anti-dsDNA levels was not observed (19).

In contrast to reported beneficial effects of BTZ in severe SLE herein, the only accomplished randomized controlled trial did not demonstrate clinical (or serological) efficacy although the number of included cases were very low (19). Nevertheless, refractory disease is not uncommon and BTZ could be one of several pharmaceutical alternatives to be considered in severe SLE resistant to conventional immunosuppressive agents. As patients with severe renal or CNS involvement, which have failed on drugs like CYC or RTX, are usually not suitable for randomized controlled trials clinicians often need to rely on empirical knowledge and off-label therapy may be required. Consequently, clinical guidance for these severe cases may be valuable. This was the rationale why we decided to retrospectively compile the available 7-years nationwide clinical experience of BTZ in SLE.

The majority of patients selected for BTZ had severe renal involvement. Based on this, it was crucial to follow renal function, i.e. eGFR, plasma albumin and the urine albumin/creatinine ratio. Interestingly, the significant beneficial effects on plasma albumin and albuminuria were slow and met statistical significance after the BTZ cycles were ended. Whether the decreasing proteinuria was associated with BTZ *per se* or with the DMARDs prescribed after BTZ cycles remain an open question. However, it is important to remember that, prior to the BTZ cycles, most patients had been resistant to the same DMARDs.

For all treatments, the level of efficacy must be accompanied by a reasonable safety profile. Side-effects during BTZ treatment were reported in as much as 50% of the patients, particularly infections. Prior studies have also observed infections as a frequent adverse event (15, 19). Pulmonary embolism and cryptogenic organizing pneumonia (COP) were the most severe reported side-effects, whereof the latter required therapeutic plasma exchange. To notice, the included patients were heavily immunosuppressed and had manifestations refractory to standard treatment regimens. Adverse events in this group of patients are not unexpected (32, 33). Nevertheless, neuropathy was not recorded in any of our patients in contrast to previous reports (15–17). Besides the clinical side-effects, we further investigated safety regarding blood cell counts and CRP. Hemoglobin was significantly decreased early, but elevated levels were observed over time and reached statistical significance at the 12-month follow-up visit. However, as shown in **Figure 3**, most safety parameters remained stable.

This was not a randomized controlled trial, and a comparator group was not included which must be taken into account. Thus, the observational nature of the data inevitably leads to selection bias. In addition, patients who responded to BTZ without significant side-effects have contributed with longitudinal data to a greater extent than those who experienced adverse events and discontinued treatment. The number of included patients were indeed low but represent a complete coverage of Swedish cases with SLE receiving BTZ during 2014−2020 and the sample size is comparable, or even larger, than previous reports (15–17). Short-time follow-up of the first two Swedish cases treated with BTZ has previously been reported but substantially longer follow-up was reported herein (20). The Swedish healthcare system’s universal access and the use of large SLE cohorts with well-characterized patients at each University unit with close follow-up by a limited number of experienced rheumatologists constitute major strengths of the study (34). Patients’ ethnicity is known to affect treatment effects in SLE. Although both Asian, African and Hispanic subjects were included herein, 7 of 12 were Caucasians. Extrapolation of therapeutic effects to different ethnicities should be done with caution.

To summarize, in a majority of patients with severe SLE manifestations irresponsive to conventional immunosuppressive agents, we observed favorable therapeutic effects of BTZ used in combination with corticosteroids. Reduction of proteinuria was seen over time as well as seroconversion of several autoantibodies. The tolerance was good in most patients, but mild adverse events was not uncommon. As BTZ may cause hypogammaglobinemia, special attention should be paid to infections.

## Data Availability Statement

The original contributions presented in the study are included in the article/supplementary material. Further inquiries can be directed to the corresponding author.

## Ethics Statement

Ethical approval was not required for this study, in accordance with the local legislation and institutional requirements, because this was an observational study evaluating treatment effects of a drug approved for another diagnosis. The study complied with the ethical principles of the Declaration of Helsinki. In Sweden, drugs are allowed to be used off label. The patients/participants provided their written informed consent to participate in this study.

## Author Contributions

Conceptualization, TW and CS. Methodology, TW, IG, and CS. Validation, TW and CS. Formal analysis, TW. Investigation, TW, IG, RH, DL, ET, PE, and CS. Data curation, TW and CS. Writing−original draft preparation, TW. Writing−review and editing, TW, IG, RH, DL, ET, PE, and CS. Visualization, TW. Supervision, CS. Project administration, TW, IG, RH, DL, ET, PE, and CS. All authors contributed to the article and approved the submitted version.

## Funding

This work was supported by grants from the Swedish Rheumatism Association, the Region Östergötland (ALF Grants and Research Grant to TW), the Gustafsson Foundation, the King Gustaf V’s 80-year Anniversary foundation and the King Gustaf V and Queen Victoria’s Freemasons foundation.

## Conflict of Interest

The authors declare that the research was conducted in the absence of any commercial or financial relationships that could be construed as a potential conflict of interest.

## Publisher’s Note

All claims expressed in this article are solely those of the authors and do not necessarily represent those of their affiliated organizations, or those of the publisher, the editors and the reviewers. Any product that may be evaluated in this article, or claim that may be made by its manufacturer, is not guaranteed or endorsed by the publisher.

## References

[1] Piga M Arnaud L. The Main Challenges in Systemic Lupus Erythematosus: Where Do We Stand? J Clin Med (2021) 10(2):243. doi: 10.3390/jcm10020243 PMC7827672 33440874

[2] Bjork M Dahlstrom O Wettero J Sjowall C. Quality of Life and Acquired Organ Damage are Intimately Related to Activity Limitations in Patients With Systemic Lupus Erythematosus. BMC Musculoskelet Disord (2015) 16:188. doi: 10.1186/s12891-015-0621-3 26264937 PMC4531389

[3] Narain S Berman N Furie R. Biologics in the Treatment of Sjogren's Syndrome, Systemic Lupus Erythematosus, and Lupus Nephritis. Curr Opin Rheumatol (2020) 32(6):609–16. doi: 10.1097/BOR.0000000000000754 33002950

[4] Atisha-Fregoso Y Toz B Diamond B. Meant to B: B Cells as a Therapeutic Target in Systemic Lupus Erythematosus. J Clin Invest (2021) 131(12):e149095. doi: 10.1172/JCI149095 PMC8203443 34128474

[5] Frodlund M Wettero J Dahle C Dahlstrom O Skogh T Ronnelid J. Longitudinal Anti-Nuclear Antibody (ANA) Seroconversion in Systemic Lupus Erythematosus: A Prospective Study of Swedish Cases With Recent-Onset Disease. Clin Exp Immunol (2020) 199(3):245–54. doi: 10.1111/cei.13402 PMC7008226 31778219

[6] Mummert E Fritzler MJ Sjowall C Bentow C Mahler M. The Clinical Utility of Anti-Double-Stranded DNA Antibodies and the Challenges of Their Determination. J Immunol Methods (2018) 459:11–9. doi: 10.1016/j.jim.2018.05.014 29807021

[7] Parodis I Stockfelt M. Sjowall C. B Cell Therapy in Systemic Lupus Erythematosus: From Rationale to Clinical Practice. Front Med (Lausanne) (2020) 7:316. doi: 10.3389/fmed.2020.00316 32754605 PMC7381321

[8] Alexander T Thiel A Rosen O Massenkeil G Sattler A Kohler S. Depletion of Autoreactive Immunologic Memory Followed by Autologous Hematopoietic Stem Cell Transplantation in Patients With Refractory SLE Induces Long-Term Remission Through De Novo Generation of a Juvenile and Tolerant Immune System. Blood (2009) 113(1):214–23. doi: 10.1182/blood-2008-07-168286 18824594

[9] Hoyer BF Moser K Hauser AE Peddinghaus A Voigt C Eilat D. Short-Lived Plasmablasts and Long-Lived Plasma Cells Contribute to Chronic Humoral Autoimmunity in NZB/W Mice. J Exp Med (2004) 199(11):1577–84. doi: 10.1084/jem.20040168 PMC2211779 15173206

[10] Benson MJ Dillon SR Castigli E Geha RS Xu S Lam KP. Cutting Edge: The Dependence of Plasma Cells and Independence of Memory B Cells on BAFF and APRIL. J Immunol (2008) 180(6):3655–9. doi: 10.4049/jimmunol.180.6.3655 18322170

[11] Neubert K Meister S Moser K Weisel F Maseda D Amann K. The Proteasome Inhibitor Bortezomib Depletes Plasma Cells and Protects Mice With Lupus-Like Disease From Nephritis. Nat Med (2008) 14(7):748–55. doi: 10.1038/nm1763 18542049

[12] Obeng EA Carlson LM Gutman DM Harrington WJ Jr Lee KP Boise LH. Proteasome Inhibitors Induce a Terminal Unfolded Protein Response in Multiple Myeloma Cells. Blood (2006) 107(12):4907–16. doi: 10.1182/blood-2005-08-3531 PMC1895817 16507771

[13] Richardson PG Sonneveld P Schuster MW Irwin D Stadtmauer EA Facon T. Bortezomib or High-Dose Dexamethasone for Relapsed Multiple Myeloma. N Engl J Med (2005) 352(24):2487–98. doi: 10.1056/NEJMoa043445 15958804

[14] Alexander T Cheng Q Klotsche J Khodadadi L Waka A Biesen R. Proteasome Inhibition With Bortezomib Induces a Therapeutically Relevant Depletion of Plasma Cells in SLE But Does Not Target Their Precursors. Eur J Immunol (2018) 48(9):1573–9. doi: 10.1002/eji.201847492 29979809

[15] Alexander T Sarfert R Klotsche J Kuhl AA Rubbert-Roth A Lorenz HM. The Proteasome Inhibitior Bortezomib Depletes Plasma Cells and Ameliorates Clinical Manifestations of Refractory Systemic Lupus Erythematosus. Ann Rheum Dis (2015) 74(7):1474–8. doi: 10.1136/annrheumdis-2014-206016 PMC4484251 25710470

[16] Segarra A Arredondo KV Jaramillo J Jatem E Salcedo MT Agraz I. Efficacy and Safety of Bortezomib in Refractory Lupus Nephritis: A Single-Center Experience. Lupus (2020) 29(2):118–25. doi: 10.1177/0961203319896018 31865857

[17] Zhang H Liu Z Huang L Hou J Zhou M Huang X. The Short-Term Efficacy of Bortezomib Combined With Glucocorticoids for the Treatment of Refractory Lupus Nephritis. Lupus (2017) 26(9):952–8. doi: 10.1177/0961203316686703 28059023

[18] van Dam LS Osmani Z Kamerling SWA Kraaij T Bakker JA Scherer HU. A Reverse Translational Study on the Effect of Rituximab, Rituximab Plus Belimumab, or Bortezomib on the Humoral Autoimmune Response in SLE. Rheumatol (Oxford) (2020) 59(10):2734–45. doi: 10.1093/rheumatology/kez623 PMC7516125 31951278

[19] Ishii T Tanaka Y Kawakami A Saito K Ichinose K Fujii H. Multicenter Double-Blind Randomized Controlled Trial to Evaluate the Effectiveness and Safety of Bortezomib as a Treatment for Refractory Systemic Lupus Erythematosus. Mod Rheumatol (2018) 28(6):986–92. doi: 10.1080/14397595.2018.1432331 29363990

[20] Sjowall C Hjorth M Eriksson P. Successful Treatment of Refractory Systemic Lupus Erythematosus Using Proteasome Inhibitor Bortezomib Followed by Belimumab: Description of Two Cases. Lupus (2017) 26(12):1333–8. doi: 10.1177/0961203317691371 28162031

[21] Hochberg MC. Updating the American College of Rheumatology Revised Criteria for the Classification of Systemic Lupus Erythematosus. Arthritis Rheum (1997) 40(9):1725. doi: 10.1002/art.1780400928 9324032

[22] Weening JJ D'Agati VD Schwartz MM Seshan SV Alpers CE Appel GB. The Classification of Glomerulonephritis in Systemic Lupus Erythematosus Revisited. Kidney Int (2004) 65(2):521–30. doi: 10.1111/j.1523-1755.2004.00443.x 14717922

[23] Batts KP Ludwig J. Chronic Hepatitis. An Update on Terminology and Reporting. Am J Surg Pathol (1995) 19(12):1409–17. doi: 10.1097/00000478-199512000-00007 7503362

[24] Griffiths B Mosca M Gordon C. Assessment of Patients With Systemic Lupus Erythematosus and the Use of Lupus Disease Activity Indices. Best Pract Res Clin Rheumatol (2005) 19(5):685–708. doi: 10.1016/j.berh.2005.03.010 16150398

[25] Gladman DD Ibanez D Urowitz MB. Systemic Lupus Erythematosus Disease Activity Index 2000. J Rheumatol (2002) 29(2):288–91. 11838846

[26] Gladman D Ginzler E Goldsmith C Fortin P Liang M Urowitz M. The Development and Initial Validation of the Systemic Lupus International Collaborating Clinics/American College of Rheumatology Damage Index for Systemic Lupus Erythematosus. Arthritis Rheum (1996) 39(3):363–9. doi: 10.1002/art.1780390303 8607884

[27] Levey AS Coresh J Greene T Stevens LA Zhang YL Hendriksen S. Using Standardized Serum Creatinine Values in the Modification of Diet in Renal Disease Study Equation for Estimating Glomerular Filtration Rate. Ann Intern Med (2006) 145(4):247–54. doi: 10.7326/0003-4819-145-4-200608150-00004 16908915

[28] Parodis I Akerstrom E Sjowall C Sohrabian A Jonsen A Gomez A. Autoantibody and Cytokine Profiles During Treatment With Belimumab in Patients With Systemic Lupus Erythematosus. Int J Mol Sci (2020) 21(10):3463. doi: 10.3390/ijms21103463 PMC7278961 32422945

[29] Sciascia S Rubini E Radin M Cecchi I Rossi D Roccatello D. Anticardiolipin and Anti-Beta 2 Glycoprotein-I Antibodies Disappearance in Patients With Systemic Lupus Erythematosus and Antiphospholipid Syndrome While on Belimumab. Ann Rheum Dis (2018) 77(11):1694–5. doi: 10.1136/annrheumdis-2018-213496 29776976

[30] Frodlund M Walhelm T Dahle C Sjöwall C. Longitudinal Analysis of Anti-Cardiolipin and Anti-β2-glycoprotein-I Antibodies in Recent-Onset Systemic Lupus Erythematosus: A Prospective Study in Swedish Patients. Front Med (Lausanne) (2021) 8:646846. doi: 10.3389/fmed.2021.646846 PMC7959716 33732724

[31] Han S Zhuang H Shumyak S Yang L Reeves WH. Mechanisms of Autoantibody Production in Systemic Lupus Erythematosus. Front Immunol (2015) 6:228. doi: 10.3389/fimmu.2015.00228 26029213 PMC4429614

[32] Falasinnu T Chaichian Y Li J Chung S Waitzfelder BE Fortmann SP. Does SLE Widen or Narrow Race/Ethnic Disparities in the Risk of Five Co-Morbid Conditions? Evidence From a Community-Based Outpatient Care System. Lupus (2019) 28(14):1619–27. doi: 10.1177/0961203319884646 PMC8459796 31660790

[33] Konig MF Kim AH Scheetz MH Graef ER Liew JW Simard J. Baseline Use of Hydroxychloroquine in Systemic Lupus Erythematosus Does Not Preclude SARS-CoV-2 Infection and Severe COVID-19. Ann Rheum Dis (2020) 79(10):1386–8. doi: 10.1136/annrheumdis-2020-217690 PMC8258626 32381561

[34] Reid S Alexsson A Frodlund M Morris D Sandling JK Bolin K. High Genetic Risk Score Is Associated With Early Disease Onset, Damage Accrual and Decreased Survival in Systemic Lupus Erythematosus. Ann Rheum Dis (2020) 79(3):363–9. doi: 10.1136/annrheumdis-2019-216227 PMC7034364 31826855

